# Positive Emotion Facilitates Audiovisual Binding

**DOI:** 10.3389/fnint.2015.00066

**Published:** 2016-01-25

**Authors:** Miho S. Kitamura, Katsumi Watanabe, Norimichi Kitagawa

**Affiliations:** ^1^Faculty of Science and Engineering, Waseda UniversityTokyo, Japan; ^2^NTT Communication Science Laboratories, NTT CorporationTokyo, Japan; ^3^Research Center for Advanced Science and Technology, The University of TokyoTokyo, Japan

**Keywords:** positive mood, depression, audiovisual, binding, multisensory

## Abstract

It has been shown that positive emotions can facilitate integrative and associative information processing in cognitive functions. The present study examined whether emotions in observers can also enhance perceptual integrative processes. We tested 125 participants in total for revealing the effects of emotional states and traits in observers on the multisensory binding between auditory and visual signals. Participants in Experiment 1 observed two identical visual disks moving toward each other, coinciding, and moving away, presented with a brief sound. We found that for participants with lower depressive tendency, induced happy moods increased the width of the temporal binding window of the sound-induced bounce percept in the stream/bounce display, while no effect was found for the participants with higher depressive tendency. In contrast, no effect of mood was observed for a simple audiovisual simultaneity discrimination task in Experiment 2. These results provide the first empirical evidence of a dependency of multisensory binding upon emotional states and traits, revealing that positive emotions can facilitate the multisensory binding processes at a perceptual level.

A number of studies have documented how multisensory binding depends on features of sensory signals such as spatial congruency, temporal synchrony, and crossmodal correspondence (for reviews, see Calvert et al., 2004; Stein, 2012). However, other studies have examined top-down influences such as attentional load (for a review, see Talsma et al., 2010), perceptual training (Powers et al., 2009), and stimulus context (Kitagawa and Spence, 2005) on multisensory binding, and their results suggest that multisensory binding depends not only on the physical characteristics of multisensory events but also on the internal states of observers. Emotion, including current moods and emotional traits (e.g., depression), has been shown to influence association processes at a cognitive level (Isen et al., 1985, 1987). However, it has not yet been addressed whether emotional states modulate multisensory binding at a perceptual level.

Positive emotions can modify perceptual information processing. For example, positive moods expand the spatial span of attention and facilitate the global processing of visual images (Gasper and Clore, 2002; Fredrickson and Branigan, 2005; Rowe et al., 2007; Soto et al., 2009). In addition, a recent study has suggested that positive emotion also broadens not only spatial attention but also temporal attention toward voluntary body movements (Rigoni et al., 2015). Because positive emotions also improve cognitive functions such as problem solving and unique word associations (Isen et al., 1985, 1987; Estrada et al., 1997), the expansion of the range of perceptual information processing by positive moods would underlie the improvement in these cognitive functions. The facilitation of associative (or integrative) processes by positive mood would be considered as a general process that is shared by multiple levels of human information processing. The previous studies focused on the effect of positive emotional states only on a single sensory modality (predominantly on vision). However, our perceptual systems encounter multisensory events in the natural environment and need to integrate or bind them. The present study aimed to examine whether observers’ positive moods would change multisensory associative (binding) processes.

A recent study examined whether prior presentation of negative emotional stimuli modulate a subsequent audiovisual binding process in the ventriloquism effect, where the perceived location of an auditory event is shifted toward a concurrently presented visual event (Maiworm et al., 2012). They showed that the magnitude of the ventriloquism effect was reduced by a threatening auditory voice presented prior to the target audiovisual stimuli. The results suggest that the stimulation with negative emotional voice might bias the access to a single sensory modality, in this case audition, resulting in the decreased audiovisual binding. Although the results imply that a neutral mood (i.e., relatively positive rather than threatened) might be a better condition for audiovisual association processing (leading to the ventriloquism effect), it is still unclear whether positive rather than neutral emotional states in individuals can modulate multisensory binding. In this study, by using the mood induction technique, we examined whether positive moods would facilitate multisensory binding. We tested this possibility in the perception of a bouncing event in a stream/bounce display (Experiment 1) and in the perception of audiovisual temporal simultaneity (Experiment 2).

In the stream/bounce display, two identical visual objects move toward each other, coincide, and then move away. The two objects are perceived as either streaming through or bouncing off each other. Although these two interpretations are mutually exclusive and theoretically ambiguous, a brief sound presented simultaneously with the visual coincidence facilitates the bounce percept (Sekuler et al., 1997; Watanabe and Shimojo, 2001). Thus, the binding of the sound and the visual coincidence disambiguates the interpretation of the visual event. This effect of audiovisual binding has been shown to decrease as the sound is presented further away in time from the visual coincidence, indicating the existence of a temporal window of audiovisual binding for the stream/bounce illusion (e.g., Watanabe and Shimojo, 2001). Importantly, it has been reported that the width of the temporal window of the sound-induced bounce percept differs from that of audiovisual simultaneity (Watanabe, 2001). We expected that if positive moods would facilitate audiovisual binding, we would find a difference between happy and non-happy moods for the width of the temporal window of audiovisual binding in the stream/bounce illusion (Experiment 1) and/or of audiovisual simultaneity between the simple audiovisual stimuli in general (Experiment 2).

We induced positive moods by letting participants hear happy-sounding music pieces of their own choice (e.g., Westermann et al., 1996; Blood and Zatorre, 2001; Zentner et al., 2008). Note that when examining the effects of current emotional states, we also have to consider the effect of individuals’ emotional traits (e.g., Verona et al., 2002). Recent research has shown that the effects of mood induction can differ between individuals due to their depressive tendencies (Horner et al., 2014; Horacek et al., 2015). Individuals with depression tendencies cannot achieve or maintain a positive mood (Horner et al., 2014), and a mood induction may have an opposite effect on the neural response to emotional stimuli between depressive and non-depressive people (Horacek et al., 2015). Therefore, in the present study, we also assessed the depression level of participants using the Beck Depression Inventory (Beck et al., 1961). We divided the participants into two groups according to their depression level and examined the effects of mood on the audiovisual binding tasks.

## Experiment 1

### Materials and Methods

#### Participants

Sixty-four participants (33 females and 31 males) took part in Experiment 1. The mean age was 26.6 years with a standard deviation (*SD*) of 6.6. Two participants (one female and one male) were excluded because their questionnaire was incomplete. All participants reported normal hearing and normal or corrected-to-normal vision. They were naïve as to the purpose of the experiment. The experiments reported here were approved by the internal review board of the Research Center for Advanced Science and Technology (The University of Tokyo) and the ethics committee of NTT Communication Science Laboratories.

The participants were paid for their participation and gave their informed consent prior to their inclusion in the experiments. In the present study, we recruited individuals without a history of mental disorder in order to avoid including individuals with clinical level depression. The participants completed the Beck Depression Inventory before the experiments (Beck et al., 1961), and on the basis of the total score, they were divided into two groups: lower-depression (a score of 9 or less) and higher-depression (a score above 9) groups. A score of 9 was used as a cut-off from minimal depression to other levels (mild, moderate, and severe depression; Beck et al., 1988). For both experiments, the average scores of the Beck depression inventory of lower- and higher-depression groups were 4.7 (*SD* = 2.7) and 14.7 (*SD* = 3.9), respectively. The maximum scores were 23 in Experiment 1 and 21 in Experiment 2. These scores in the higher-depression group were within the range of mild-to-moderate depression. Note that the higher and lower depression levels were all relative within the participants of the present study.

#### Apparatus, Materials and Procedure

The experiments were conducted in a sound-attenuated and dimly lit chamber. The participants sat at a table, and their head was fixed with a chin-and-forehead rest. Visual stimuli were presented on a CRT display (60 Hz refresh) located at a distance of 57 cm. Auditory stimuli were presented to participants through open headphones (Sennheiser, HD600). The experiment was controlled and the data were collected by MATLAB^®^ (The MathWorks) with the Psychophysics toolbox extension (Brainard, 1997; Pelli, 1997) on an Apple Mac mini computer. To induce a happy mood, the participants were each asked to bring music pieces amounting to 20 min that made them feel happy (happy-sounding music). They were free in their choice of musical style, and indeed, the chosen materials varied widely between participants. Pink noise was used as a control sound for the non-happy condition. The happy-sounding music or pink noise was presented throughout the experimental block from a portable music player (Bose SoundDock Series III digital music system) placed on the floor behind the participants at a distance of 90 cm.

**Figure 1:**
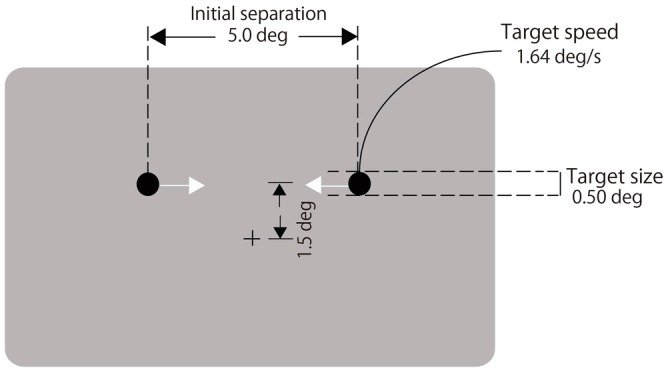
**Schematic illustration of the stream/bounce display used in Experiment 1**.

A black fixation cross (0.4° in visual angle) was displayed at the center of a gray background (72.3 cd/m^2^, see Figure 1). Two black disks (1.5 cd/m^2^, 0.5° in diameter) moved horizontally on a trajectory located 1.5° above the fixation cross. They were initially separated by 5°. Immediately after the disk appeared, they moved laterally at a rate of 1.64°/s toward each other, coincided, continued moving until each reached the other’s starting point and then disappeared (Figure 1). A 2000-Hz auditory click of 8-ms duration was presented with various stimulus onset asynchronies (SOAs) relative to the visual coincidence (−300, −225, −150, −75, 0, 75, 150, 225, 300 ms, and no click; negative values indicate the click led the visual coincidence). Participants fixated on the cross throughout each trial and observed the two moving disks. They reported whether the two disks appeared to stream through or bounce off each other by pressing either the left or right buttons. They performed 20 trials for each of the nine SOAs and the no-click condition. The order of the trials was randomized. The experiment was divided into two blocks of 100 trials. After the first block, the participants took a short break. Each block lasted for approximately 5 min.

During the experiment, including while the participants were given instructions about their task, the happy-sounding music or pink noise was presented at around 60 dBA (happy or non-happy condition; between-participants variable). The auditory click of the stream/bounce task was presented through the open-type headphones so that they could hear the happy-sounding music and pink noise, yet still easily dissociate the auditory click from the background music and noise as they were presented from the different location. Each participant’s mood (valence and arousal) was assessed using the 9-point scale at the beginning of the experiment (before listening to the happy-sounding music or the pink noise) and immediately after the stream/bounce task.

#### Analysis

The data of each participant were fitted with a Gaussian equation with the maximum likelihood estimation method:

$$pinc\;\text{exp}\left\{-\frac{{\left(SOA-\mu \right)}^{2}}{2\sigma^{2}}\right\}+pbase$$

For each participant, we obtained the mean (μ), which corresponds to the SOA yielding the maximum proportion of the bounce percept, the *SD* of the curve (*σ*), which reflects the width of the temporal binding window within which the auditory click induced the bounce percept, the lower asymptote (*pbase*) of the curve which reflects the proportion of the bounce percept with a minimum effect of audiovisual binding between the visual coincidence and auditory click, and the increment in the proportion of the bounce percept (*pinc*) from the *pbase* to the maximum proportion that corresponds to the magnitude of the effect of the audiovisual binding. Data from 10 participants whose data did not fit well to the Gaussian function (*R*^2^ < 0.63) or resulted in too small estimated values of *σ* (< 5 ms) were discarded. After this exclusion, the mean *R*^2^ was 0.869 with a *SD* of 0.09. The resulted numbers of the participants were eleven for both the happy and non-happy conditions in the higher-depression group, and fifteen for both the happy and non-happy conditions in the lower-depression group.

## Results and Discussion

### Mood Assessment

The mood manipulation by presenting the happy-sounding music succeeded in inducing happy moods in the participants. The ratings on valence and arousal were analyzed by using three-way mixed analysis of variance (ANOVA) with the induced mood (happy and non-happy) and depression level (lower and higher) as between-participant factors, and the time of measurement (pre- and post-test) as a within-participant factor. The analyses showed the significant main effects of the induced mood for valence (*F*_(1,48)_ = 4.38, *p* < 0.05, $\eta_{p}^{2}$ = 0.06) and of the time of measurement for arousal (*F*_(1,48)_ = 4.7, *p* < 0.05, $\eta_{p}^{2}$ = 0.04). Importantly, interactions between induced mood and time of measurement for both valence and arousal scores were significant (*F*_(1,48)_ = 34.44, *p* < 0.0001, $\eta_{p}^{2}$ = 0.17 for valence score and *F*_(1,48)_ = 34.72, *p* < 0.0001, $\eta_{p}^{2}$ = 0.17 for arousal score), indicating that the presentation of the happy-sounding music made the participants feel significantly more positive and more aroused (*p* < 0.001; Tukey’s Honestly Significant Difference test) than hearing the pink noise regardless of their depression level (Table 1). We found no significant differences between the happy and non-happy conditions in the pre-test ratings for both arousal and valence scores (all *p*’s > 0.05). Neither the main effect of the depression level nor interactions between the depression level and the other factors were significant for the valence and arousal scores (all *F*’s < 1).

**Table 1:** **Mean mood rating values of pre and post-test in Experiments 1 and 2**.

	Depression	Mood	Valence		Arousal	
			Pre	Post	Pre	Post
Experiment 1	Higher	Happy	5.09 (1.45)	6.64 (0.92)	4.18 (1.83)	5.90 (1.58)
		Non-happy	5.45 (1.69)	4.45 (2.07)	5.18 (2.14)	4.06 (2.17)
	Lower	Happy	5.87 (1.30)	6.47 (1.06)	4.13 (1.13)	6.07 (1.16)
		Non-happy	6.40 (1.12)	4.93 (1.38)	4.53 (1.55)	3.93 (1.53)
Experiment 2	Higher	Happy	5.25 (1.42)	6.5(1.00)	4.08 (1.51)	5.08 (2.02)
		Non-happy	5.2(1.32)	4.8(0.92)	3.3(1.03)	4.47 (1.34)
	Lower	Happy	6.25 (1.16)	6.95 (0.60)	4.15 (1.73)	5.45 (1.85)
		Non-happy	6.13 (1.36)	5.53 (1.41)	4.47 (1.19)	3.13 (1.50)

*Standard deviations are given in parentheses*.

### Stream/Bounce Task

**Figure 2:**
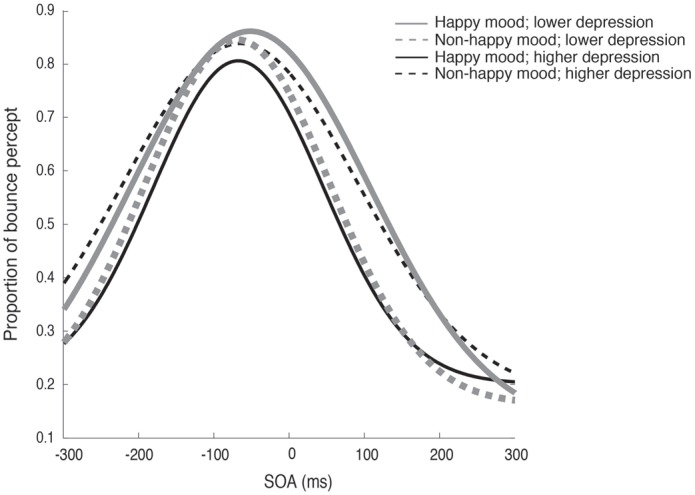
**Fitted curves depicted using the mean values of each parameter (σ, μ, *pinc* and *pbase*) that were derived from the Gaussian fitting for the happy and non-happy conditions in the two depression groups in Experiment 1**.

**Figure 3:**
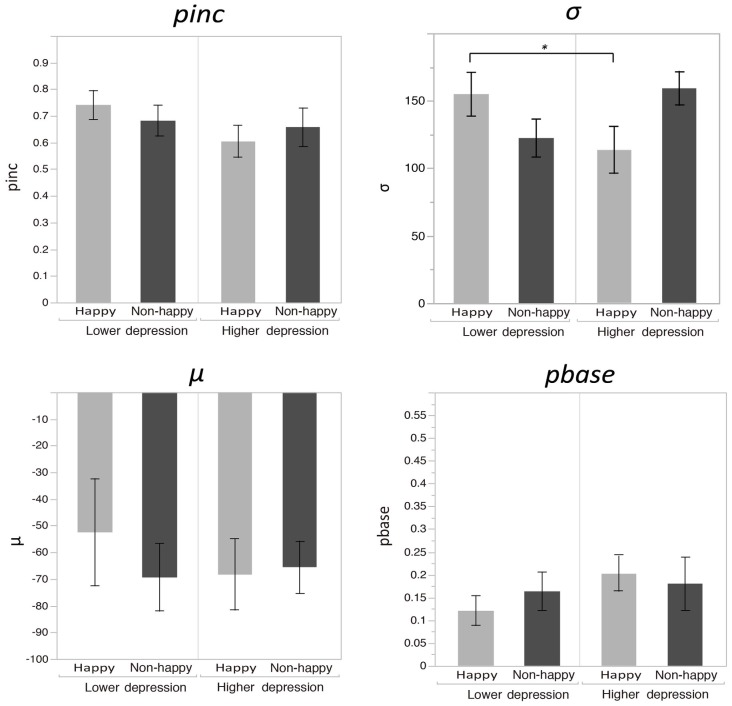
**Mean values of each parameter (σ, μ, *pinc* and *pbase*) that were derived from the Gaussian fitting for the happy and non-happy conditions in the two depression groups.** Error bars show the standard errors of the means.

Figure 2 plots the fitted curves depicted using the mean values of each parameter (σ, μ, *pinc* and *pbase*) that were derived from the Gaussian fitting for the happy and non-happy conditions in the two depression groups. All of the fitted functions show that, when the visual coincidence and the auditory click were presented close in time, the participants reported bouncing responses more often, and the number of bouncing responses decreased as they were separated in time. There was a no significant difference in mean values between the *pbase* (*M* = 0.175) and the proportion of the bounce percept in the no-sound condition (*M* = 0.162; *t*_(51)_ = 0.562, *p* = 0.57, two-tailed). The SOA inducing the peak of the functions was slightly biased toward the audio-lead direction, replicating the results of previous studies (e.g., Watanabe and Shimojo, 2001). Visual inspection of these functions showed that the width and the peak of the functions differed across the mood conditions in the two depression groups. Each parameter of the fitted function (σ, μ, *pinc* and *pbase*) was analyzed by a two-way ANOVA with the induced mood (happy and non-happy) and the depression level (higher and lower) as between-participant factors. We found significant differences only for *σ*, which reflected the width of the temporal binding window of the visual coincidence and the auditory click, whereas we found neither significant main effects of the two factors nor interactions between them in μ, *pinc* and *pbase* (all *p*’s > 0.10). The analysis for σ revealed a significant interaction between the induced mood and depression level (*F*_(1,48)_ = 6.93, *p* = 0.011, $\eta_{p}^{2}$ = 0.13; see Figure 3). *Post hoc* comparisons with the simple main effect analysis showed that the proportion of the bounce percept of the lower-depression group was significantly higher than that of the higher-depression group in the happy condition (*p* = 0.034). The main effects of both mood and depression were not significant (all *F*’s < 1).

**Figure 4:**
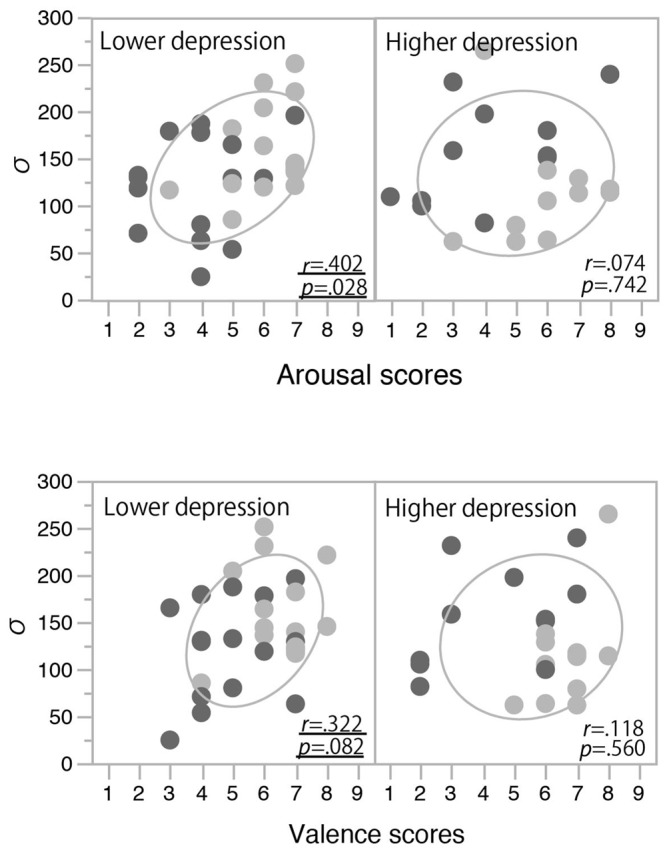
**Correlations between mood rating scores and *σ* values in Experiment 1.** Light and dark gray plots indicate happy and non-happy conditions, respectively. Ellipses show a probability ellipse of 68%.

In order to clarify the significant interaction between the induced mood and depression level observed for the temporal binding window, we examined the correlation between the mood rating scores (post-test valence and arousal scores) and *σ* values. In each panel of Figure 4, the data from the happy condition group (indicated with light gray) are located more to the right side than those from the non-happy condition group (indicated with dark gray). This means that the valence and arousal scores increased with the happy-mood induction in both the lower- and higher-depression groups. In the low-depression group, *σ* values also increased in association with those valence and arousal scores (see the left panels of Figure 4), resulting in a significant positive correlation (Pearson correlation coefficient) between the arousal scores and *σ* values (*r*_(28)_ = 0.40, *p* = 0.028) and a marginally significant correlation between the valence scores and *σ* values (*r*_(28)_ = 0.32, *p* = 0.083). We also found a significant correlation between the arousal and valence scores (*r*_(50)_ = 0.571, *p* < 0.0001), suggesting that the arousal and valence scores were not independent, but rather interacting or integrated. To confirm this notion, we calculated the multiplication of those scores (arousal scores × valence scores) and calculated the correlation coefficient between the multiplication scores and *σ* values. The multiplication scores showed a stronger correlation with *σ* values (*r*_(28)_ = 0.43, *p* < 0.018) compared to the correlations of arousal and valence scores. In other words, a more aroused and more positive mood (e.g., excited mood) seemed to facilitate the audiovisual binding more strongly. In contrast, in the higher-depression group, as the higher arousal and valence scores were not associated with an increase in *σ* values, we found no significant correlations between the mood rating scores and the width of the temporal binding window (see the right panels of Figure 4). The results suggest that a happy (and active) mood facilitates the audiovisual binding in the stream/bounce display only for the participants with relatively lower depressive traits.

The results supported our prediction that a positive mood would enhance audiovisual binding (at least for the lower-depression group). The broader temporal binding window observed in Experiment 1 might reflect decreased sensitivity to the temporal disparity (a wider temporal binding window) between auditory and visual events in general, since the sound-induced bounce percept might depend on subjective simultaneity between audiovisual signals (e.g., Kitagawa and Kitamura, 2014). We examined this possibility by using a simultaneity discrimination task in Experiment 2.

## Experiment 2

### Method

Sixty-one participants (33 females and 28 males) took part in Experiment 2. The mean age was 25.9 years with a *SD* of 6.9.

The apparatus was the same as in Experiment 1. A pair consisting of a visual flash of the disk at the center of the display lasting for 16.7 ms and the auditory click (both were identical to those used in Experiment 1) was presented twice. In one pair the flash and click were presented synchronously, whereas in the other pair they were presented with an SOA randomly chosen from the nine SOAs (−300, −225, −150, −75, 0, 75, 150, 225 and 300 ms). In half of the trials the synchronous pair of the flash and click was presented first, and in the other half it was presented second. The participants fixated on the center cross throughout each trial and indicated which pair of the flash and click was synchronous. They performed 20 trials for each of the nine SOAs. The order of the trials was randomized. The experiment was divided into two blocks of 90 trials. After the first block, the participants took a short break. Each block lasted for approximately 5 min. The mood induction procedure was identical to that in Experiment 1.

The same Gaussian function as Experiment 1 was fitted to the correct probability of the discrimination task for each participant. As the data were U-shaped, the resulting functions were also U-shaped with negative *pincs*. Data from four participants were discarded with the same criterion as Experiment 1. After this exclusion, the mean *R*^2^ was 0.831 with a *SD* of 0.12. The resulted numbers of the participants were eleven for both the happy and non-happy conditions in the higher-depression group, and in the lower-depression group, twenty-one for the happy condition and fourteen for the non-happy condition.

## Results and Discussion

### Mood Assessment

The ratings on valence and arousal were analyzed by using a three-way mixed ANOVA with the induced mood (happy and non-happy) and depression level (lower and higher) as between-participant factors, and the time of measurement (pre- and post-test) as a within-participant factor (Table 1). The analyses showed significant main effects of induced mood (for valence: *F*_(1,53)_ = 8.79, *p* < 0.01, $\eta_{p}^{2}$ = 0.21; for arousal: *F*_(1,53)_ = 7.55, *p* < 0.01, $\eta_{p}^{2}$ = 0.46). As expected, hearing the happy-sounding music made the participants feel significantly more positive and more aroused (*p* < 0.001; Tukey’s Honestly Significant Difference test) than hearing the pink noise regardless of their depression level (Table 1), resulting in significant interactions between the induced mood and the time of measurement for both valence and arousal scores (*F*_(1,53)_ = 22.46, *p* < 0.001, $\eta_{p}^{2}$ = 0.17 for valence score and *F*_(1,53)_ = 21.25, *p* < 0.0001, $\eta_{p}^{2}$ = 0.47 for arousal score). There was no significant difference between the happy and non-happy conditions before the task for either the valence and arousal scores (all *p*’s > 0.05). We also found a significant main effect of depression tendency for the valence scores (*F*_(1,53)_ = 7.92, *p* < 0.01, $\eta_{p}^{2}$ = 0.19), but not for the arousal scores (*F* < 1).

### Simultaneity Discrimination Task

**Figure 5:**
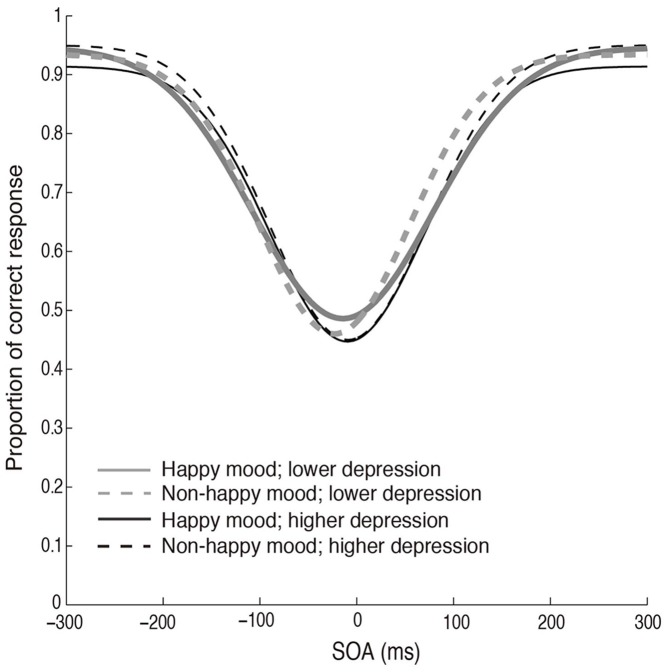
**Fitted curves depicted using the mean values of each parameter (σ, μ, *pinc* and *pbase*) that were derived from the Gaussian fitting for the happy and non-happy conditions in the two depression groups in Experiment 2**.

**Figure 6:**
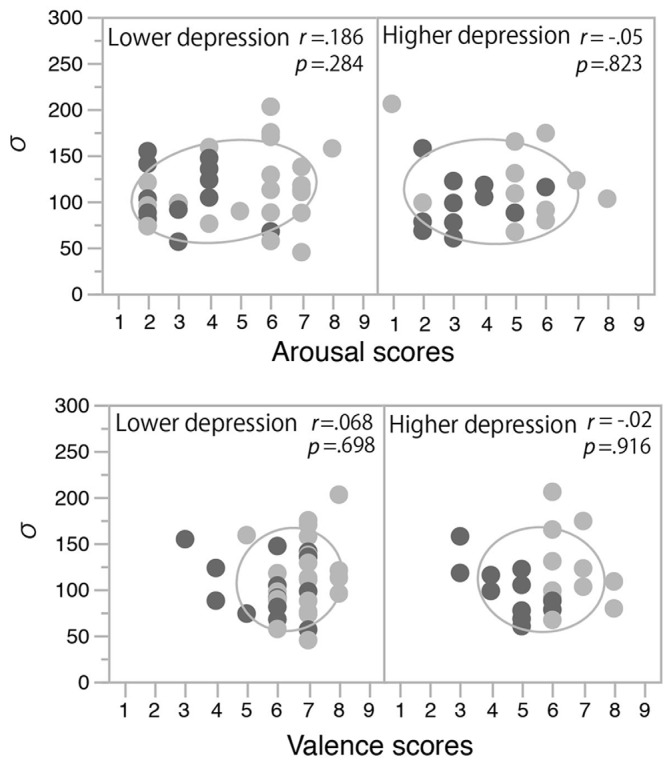
**Correlations between mood rating scores and *σ* values in Experiment 2.** Light and dark gray plots indicate happy and non-happy conditions, respectively. Ellipses show a probability ellipse of 68%.

The fitted functions depicted by the mean values of each parameter (*σ*, *μ, pinc*, and *pbase)* that were derived from the Gaussian fitting in the two mood conditions in the two depression groups are shown in Figure 5. The discrimination performance was worse when the flash and click were presented close in time, and the performance increased as the SOA increased. The fitted functions were similar across the happy and non-happy conditions in the two depression groups. Each parameter was analyzed by a two-way ANOVA with the factors of the induced mood (happy and non-happy moods) and the depression level (higher and lower). We found neither significant main effects nor interaction for all the parameters (all *p*’s > 0.05). We also found no significant correlations between the mood ratings and *σ* values (Figure 6). Thus, the results of Experiment 2 indicate that the positive mood induced by the happy-sounding music did not affect the audiovisual simultaneity perception. The results of Experiment 2, therefore, suggest that the enhancement of audiovisual binding in the stream/bounce illusion (i.e., the increased the width of audiovisual binding window) in the happy condition in Experiment 1 was not due to a reduced sensitivity of audiovisual temporal disparity. Rather, induced positive moods seemed to affect the association process in audiovisual perception.

## General Discussion

The present study examined whether a positive mood can modulate audiovisual binding, as assessed by the stream/bounce illusion and the audiovisual simultaneity discrimination performance. In Experiment 1, the positive moods induced by the happy-sounding music broadened the width of the audiovisual temporal binding window for the bounce percept. The critical finding is that the enhancement of the bounce percept positively correlated with the arousal and valence ratings. The results suggest that presenting the happy-sounding music can facilitate the audiovisual binding process. However, this was the case only for the participants with a relatively low depression level. In addition, Experiment 2 showed that the sensitivity to audiovisual asynchrony between the simple auditory and visual events (i.e., the auditory click and visual flash) was not influenced by induced moods. The present results are the first to show that positive moods can facilitate the audiovisual binding process and that this facilitation depends on the participant’s depression trait.

The happy-sounding music and the pink noise differed significantly in terms of various acoustic parameters. Therefore, it is possible that the happy-sounding music by itself caused more frequent bounce percepts or that the happy-sounding music made the click sound more salient. However, in general, a transient auditory event should be more salient when it is presented in a temporally stable background than when it is presented with temporally dynamic sounds (e.g., Moore, 2012). Thus, the temporal dynamics of the happy-sounding music would deteriorate the perceptual saliency of the auditory click more than the pink noise would, and therefore would be expected to reduce the effect of the auditory click in the stream/bounce illusion, but this expectation was opposite to what we observed in Experiment 1. Also, in Experiment 2, discrimination performance in the happy condition was no worse than that in the non-happy condition, suggesting that the audibility of the auditory click did not differ between the mood conditions. Introspective reports from some of the participants in both experiments also indicated that they had no difficulties in dissociating the auditory click from the background music. Furthermore, the enhancement of the bounce percept by the auditory click correlated with the arousal and valence levels (see Figure 4). These results suggest that induced positive moods in participants facilitated the audiovisual binding between the visual coincidences and the auditory click, resulting in the broader temporal binding window for the sound-induced bounce percept.

Maiworm et al. (2012) reported that presenting auditory stimuli having negative valence weakened audiovisual binding in the ventriloquism effect, suggesting that exogenous emotional responses modulate subsequent audiovisual bindings. Yet it was still unclear whether endogenous emotions (i.e., emotional state and emotional trait) also modulate audiovisual binding. In the present study, we found that both emotional states and emotional traits of individuals modulate the width of the audiovisual temporal binding window in the stream/bounce display. While the internal states of observers such as attentional load (for a review, see Talsma et al., 2010), perceptual training (Powers et al., 2009), and stimulus context (Kitagawa and Spence, 2005) have been shown to modulate multisensory associations, our results have firstly shown that emotional states and emotional traits also play a role in the audiovisual association process. Positive emotion has been shown to facilitate our cognitive association processes such as unique word associations and creative problem solving (Isen et al., 1985, 1987; Estrada et al., 1997). The present results showed that positive emotion would also enhance associative or integrative processing at the perceptual level. The enhancement of perceptual association by positive emotions may serve as a perceptual basis for associative cognitive processes.

While induced happy moods facilitated the audiovisual binding in the stream/bounce illusion (Experiment 1), the sensitivity to asynchrony between the simple audiovisual events was not deteriorated by induced happy moods (Experiment 2). Thus, the results suggest that the effects of emotional state on audiovisual binding may vary according to the level of binding processes. Emotional state would not affect the lower-level audiovisual association that may be seen in audiovisual temporal fusion between simple auditory and visual events. Rather, emotional state would affect higher-level audiovisual association including perceptual assessment of causal relationship between audiovisual events, as seen in the stream/bounce illusion. Donohue et al. (2015) recently reported that the influence of attention on multisensory binding depends on stimulus complexity and task demand. In future research, it would be interesting to examine how differently emotion and attention influence multisensory processing.

The correlation of the temporal binding window with the arousal level was stronger than that with the valence level. In the present study, it is difficult to separate completely the effects of attention and arousal levels. There is a possibility that higher arousal level would cause more attention to the stimuli, resulting in the facilitated audiovisual binding in the stream/bounce illusion. However, Donohue et al. (2015) have shown that more attention to the stream/bounce display narrows the temporal binding window of the audiovisual binding. If a stronger attentional focus towards the audiovisual stimuli induced by the higher arousal level had an important role in our results, the temporal binding window would decrease. However, the results were opposite. On the basis of previous studies showing the effect of positive emotion on attention (Gasper and Clore, 2002; Fredrickson and Branigan, 2005; Rowe et al., 2007; Soto et al., 2009) and our results, we would suggest that attention would be broadened by positive moods, resulting in facilitation of audiovisual integration.

The effects of induced happy moods on the audiovisual binding observed in Experiment 1 depended on the depression level of the participants. Although both the higher- and lower-depressive individuals reported more positive and more aroused moods after the happy-mood induction, we found that the induced happy mood broadened the temporal binding window for the stream/bounce illusion in the lower-depression group (i.e., the significant correlation between the mood ratings and the width of the temporal window) but not in the higher-depression group. It has been reported that emotional state can differ depending on the depression level of individuals. Individuals with higher depression cannot achieve or maintain a positive mood (Horner et al., 2014), and induced sad moods would have an opposite effect on amygdala neural responses to emotional stimuli between depressive and non-depressive individuals (Horacek et al., 2015). In light of these previous findings, even though the participants with higher depression in Experiment 1 reported more aroused and more positive moods, their moods may still not be strong enough to facilitate the audiovisual binding in the stream/bounce illusion. However, it should be noted that our participants in the higher-depression group did not include clinically severe depressive participants. In future research, it would be important to examine how depressive severity would influence audiovisual binding processes.

In conclusion, we examined whether emotional factors would modulate the audiovisual binding process by using the stream/bounce illusion and the simultaneity discrimination task. Although all the participants observed the same stream/bounce display, the participants with relatively low depression, when a positive mood was induced by music of their own choice, tended to report the bounce percept more frequently. In contrast, we did not find any emotional effects on the simultaneity discrimination task. These results suggest that the audiovisual binding processing is affected by emotional state and emotional traits. Future research will reveal the intricate nature of interaction between emotion and integration processes.

## Author Contributions

MSK, KW and NK designed the study, performed the experiments, analyzed the results and wrote the manuscript. All authors approved the final version of the manuscript.

## Conflict of Interest Statement

The authors declare that the research was conducted in the absence of any commercial or financial relationships that could be construed as a potential conflict of interest.
